# Chronic HBV infection among pregnant women and their infants in Shenyang, China

**DOI:** 10.1186/1743-422X-10-17

**Published:** 2013-01-07

**Authors:** Yang Ding, Qiuju Sheng, Li Ma, Xiaoguang Dou

**Affiliations:** 1 Department of Infectious Diseases, Shengjing Hospital of China Medical University, Shenyang, 110004, China

**Keywords:** HBV, Infection, Pregnancy, Mother to child transmission

## Abstract

**Background:**

The main transmission route of the hepatitis B virus (HBV) is mother to child transmission and contributes significantly to chronic HBV infection. Even though immunoprophylaxis with hepatitis B immunoglobulin (HBIG) and hepatitis B vaccine is administrated to neonates whose mothers are hepatitis B surface antigen (HBsAg) positive, about 10% of the neonates suffer from HBV infection in their early life.

**Objectives:**

To survey chronic HBV infection among pregnant women and their infants and analyze the reason for immunoprophylaxis failure.

**Methods:**

Serum HBsAg was tested in all pregnant women. HBVDNA and other serum HBV markers including hepatitis B e antigen (HBeAg), hepatitis B core antibody (anti-HBc) and hepatitis B surface antibody (anti-HBs) were tested among HBsAg positive pregnant women. All infants whose mothers were HBsAg positive were vaccinated with a standard immunoprophylaxis. Serum HBV markers and HBVDNA were tested among these infants at 7 months of age. HBV genotypes were analyzed among the infants and pregnant women who were HBVDNA positive.

**Results:**

The prevalence of HBsAg, anti-HBc and anti-HBs among 4,536 pregnant women was 5.49%, 29.65% and 58.55%, respectively. The prevalence of HBsAg, anti-HBc and anti-HBs among pregnant women older than 20 years of age was significantly different compared to pregnant women younger than 20 years of age (4.54, 5.69 and 0.61 times, prevalence older vs. younger, respectively. *P*<0.05, 0.01, 0.05, respectively). Among 249 HBsAg positive pregnant women, 167 (67.07%) were HBeAg positive, 204 (81.93%) were HBVDNA positive and only 37 (14.86%) had HBVDNA >10^7^ IU/ml. Among the infants whose mothers were HBsAg positive, 214 (85.94%) infants were anti-HBs positive. There were 12 (4.82%) infants who were HBsAg and HBVDNA positive, and all 12 of these infants mothers were HBeAg positive and had HBVDNA >10^7^ IU/ml. Genotypes B and C were present among 165 pregnant women and genotype C was present in 85 pregnant women. There were 12 infants who were HBsAg positive and had the same HBV genotypes as their mothers. There was a significant difference in genotypes between the pregnant women whose infants were infected with HBV compared to those without HBV infection (P < 0.05).

**Conclusions:**

There was a significant decline in HBsAg prevalence among pregnant women and their infants in Shenyang. Genotype C might be a risk factor for mother to child transmission of HBV.

## Background

Worldwide, there are about 350 million people with chronic hepatitis B virus (HBV) infection and about one million deaths per year due to chronic hepatitis, cirrhosis, and hepatocellular carcinoma [1,2]. Mother to child transmission of HBV is the main transmission route and contributes significantly to chronic HBV infection [3].

China used to be an area of HBV high epidemicity. About 10% of infants whose mothers were HBV positive suffered from HBV infection in their early life. It has been reported that the prevalence of Hepatitis B surface antigen (HBsAg) has been reduced from 9.75% in 1992 to 7.18% in 2006 [4]. In our study, we aimed to survey chronic HBV infection among pregnant women and their infants in Shenyang, China and analyze the reason for immunoprophylaxis failure.

### Subjects and methods

Between June 2010 and November 2011, all pregnant women who delivered at Shengjing Hospital of China Medical University were enrolled. Because immunoprophylaxis with hepatitis B immunoglobulin (HBIG) and hepatitis B vaccine has been universally administrated in Shenyang since 1990, the pregnant women were divided into two age groups: younger than 20 years and older than 20 years of age. Serum HBsAg was tested in all pregnant women. HBVDNA and other serum HBV markers including hepatitis B e antigen (HBeAg), hepatitis B core antibody (anti-HBc) and hepatitis B surface antibody (anti-HBs) were tested among HBsAg positive pregnant women.

All infants whose mothers were HBsAg positive were vaccinated with hepatitis B vaccine 20 ug (Changchun Biotech Research Institute, Changchun, China) according to a standard vaccination regimen (within 12 h of birth, at week 4, and week 24) and 200 IU doses of HBIG (Hualan Biotech Research Institute, Xinxiang, China) immediately (within 2 h of birth) and at day 15. These infants were followed up at 7 months of age. Serum HBV markers and HBVDNA were tested. HBV genotypes were tested among the infants and pregnant women who were HBVDNA positive.

HBV markers were quantitatively assayed with a chemiluminescent microparticle immunoassay using an automated Abbott AxSYM analyser (Abbott, USA). HBsAg below 0.05 IU/ml, anti-HBs below 10.0 mIU/ml, HBeAg and anti-HBc below the signal/cutoff ratio of 1.0 were considered negative. HBVDNA was analyzed using a real-time PCR assay using COBAS AmpliPrep/COBAS TaqMan 48 analyser (Roche, Switzerland), HBVDNA<12 IU/ml was considered negative. HBV genotypes were analyzed using the TRUGENE HBV genotyping kit (Siemens medical solutions diagnostics, Saint Denis, France).

Statistical analysis was performed using the Statistical Package for the Social Sciences (version 17.0, SPSS, Chicago, IL, USA). Data were analyzed by Fisher’s exact test and calculated odds ratio (OR) value and 95% confidence interval (CI). Statistically significant differences were defined as P<0.05.

## Results

### The prevalence of HBsAg, anti-HBc and anti-HBs among pregnant women

A total of 4,536 pregnant women aged 16 – 45 years were screened for HBsAg. The prevalence of HBsAg among pregnant women was 5.49% (249/4536). The prevalence of HBsAg among pregnant women older than 20 years (5.64%) was 4.54 times that among pregnant women younger than 20 years (1.30%) (*OR* = 4.54, 95% CI: 1.12~18.43). This higher prevalence of HBsAg among pregnant women older than 20 years was significant higher compared to pregnant women younger than 20 years (P < 0.05) (Table 1).

**Table 1:** The prevalence of HBsAg, anti-HBc and anti-HBs among pregnant women in the different age groups

**Age group**	**HBsAg**		**OR**	**anti-HBc**		**OR**	**anti-HBs**		**OR**
	**Positive**	**Negative**		**Positive**	**Negative**		**Positive**	**Negative**	
less than 20 years old	2	152	1.0^@^	11	143	1.0^@^	107	47	1.0^@^
n=154	(1.30%)	(98.70%)		(7.14%)	(92.86%)		(69.48%)	(30.54%)	
older than 20 years	247	4135	4.54	1334	3048	5.69	2549	1833	0.61
n=4382	(5.64%)	(94.36%)		(30.44%)	(69.56%)		(58.17%)	(41.83%)	
total	249	4287		1345	3191		2656	1880	
n=4536	(5.49%)	(94.51%)		(29.65%)	(70.35%)		(58.55%)	(41.45%)	
X^2^	4.59			37.61			7.38		
*P*	< 0.05			< 0.01			< 0.05		

@-reference category.

The prevalence of anti-HBc among pregnant women was 29.65% (1345/4536). The prevalence of anti-HBc among pregnant women older than 20 years (30.44%) was 5.69 times that among pregnant women younger than 20 years (7.14%) (*OR* = 5.69, 95%CI: 3.07~10.54). This higher prevalence of anti-HBc among pregnant women older than 20 years was significant higher compared to pregnant women younger than 20 years (P < 0.01) (Table 1).

The prevalence of anti-HBs among pregnant women was 58.55% (2656/4536). The prevalence of anti-HBs among pregnant women older than 20 years (58.17%) was 0.61 times that among pregnant women younger than 20 years (69.48%) (*OR* = 0.61, 95%CI: 0.43~0.87). This lower prevalence of anti-HBs among pregnant women older than 20 years was significant lower compared to pregnant women younger than 20 years (P < 0.05) (Table 1).

### HBeAg status and HBVDNA load among HBsAg positive pregnant women

HBeAg and HBVDNA were analyzed among the 249 HBsAg positive pregnant women. Of the 249 women, 167 (67.07%) were HBeAg positive and 204 (81.93%) were HBVDNA positive. Of the 204 HBVDNA positive women, only 37 (14.86%) had HBVDNA >10^7^ IU/ml. All pregnant women with HBVDNA >10^7^ IU/ml were HBeAg positive and all those who were HBVDNA negative were HBeAg negative (Table 2).

**Table 2:** HBeAg status and HBVDNA load among HBsAg positive pregnant women

**HBVDNA**	**HBeAg positive**	**HBeAg negative**	**Total**
**IU/ml**	**n=167**	**n=82**	**n=249**
<12	0(0%)	45(54.88%)	45(18.07%)
(12–50)	2(1.20%)	9(39.02%)	11(4.42%)
(50-<10^7^)	128(76.64%)	28(6.10%)	156(62.65%)
(>10^7^)	37(22.16%)	0(0%)	37(14.86%)

### HBV infection of infants

There were 249 infants whose mothers were HBsAg positive and were vaccinated with standard immunoprophylaxis and followed up at 7 months of age. There were 214 (85.94%) infants who tested anti-HBs positive only. There were 12 (4.82%) infants who were HBsAg and HBVDNA positive, and their mothers were HBeAg positive and HBVDNA >10^7^ IU/ml (Table 3). There were 2 (16.67%) infants who were anti-HBs positive among the 12 HBsAg positive infants.The anti-HBs titers were 547.25 mIU/ml and 1224.58 mIU/ml, respectively.

**Table 3:** Serological and virological profile for the 12 infected mother-infant pairs

**No.**	**Mothers**					**Infants**				
	**HBsAg**	**HBeAg**	**anti-HBc**	**anti-HBs**	**HBVDNA (IU/ml)**	**HBsAg**	**HBeAg**	**anti-HBc**	**anti-HBs**	**HBVDNA (IU/ml)**
1	+	+	+	-	5.56×10^7^	+	+	+	-	1.14×10^7^
2	+	+	+	-	5.49×10^8^	+	+	+	+	1.73×10^7^
3	+	+	+	-	2.37×10^8^	+	+	+	-	2.80×10^8^
4	+	+	+	-	4.12×10^8^	+	+	+	+	2.21×10^7^
5	+	+	+	-	2.89×10^9^	+	+	+	-	9.63×10^8^
6	+	+	+	-	2.16×10^7^	+	+	+	-	6.98×10^7^
7	+	+	+	-	2.63×10^8^	+	+	+	-	2.94×10^7^
8	+	+	+	-	2.03×10^8^	+	+	+	-	7.82×10^7^
9	+	+	+	-	6.61×10^7^	+	+	+	-	2.24×10^7^
10	+	+	+	-	7.17×10^7^	+	+	+	-	1.15×10^7^
11	+	+	+	-	1.93×10^7^	+	+	+	-	3.77×10^7^
12	+	+	+	-	2.59×10^7^	+	+	+	-	6.42×10^7^

### HBV genotypes among pregnant women and their infants

Only genotypes B and C had been found among 165 pregnant women (Table 4). The 165 pregnant women were divided into two groups on the basis of their infants being with or without HBV infection. There were 12 infants who were HBsAg positive and had the same HBV genotypes as their mothers. Genotype C was present in 85 pregnant women. Genotype C was present in 10 pregnant women whose infants were infected with HBV and 75 pregnant women whose infants were not infected with HBV. Genotype B was present in 2 pregnant women whose infants were infected with HBV. There was a significant difference between pregnant women whose infants were infected with HBV and those without HBV infection (P=0.04 < 0.05). Genotype C was present in 2 anti-HBs and HBsAg positive infants.

**Table 4:** HBV Genotypes among 165 pregnant women

**Group**	**Genotype B**	**Genotype C**
Infants with HBV infection	**2**	**10**
n=12		
Infants without HBV infection	**78**	**75**
n=153		
total	**80**	**85**
n=165		

χ^2^ =3.96, *P*< 0.05.

## Discussions

There are more than 130 million chronic HBV carriers in China, 30%-50% of whom are thought to have acquired HBV infection from mother-to-child transmission [5-7]. Preventing the mother-to-child transmission route can significantly decrease HBV infection among infants and can relieve the HBV disease burden. In China, 90% of HBV mother-to-child transmission can be prevented by the administration of immunoprophylaxis with HBIG and the hepatitis B vaccine [8,9]. It has been confirmed that the failure of immunoprophylaxis is mainly associated with maternal seropositivity for HBeAg and high viral load [10-12]. It is recommended that HBsAg-positive pregnant women who are HBeAg positive and have a high viral load be given antivirals in late pregnancy to suppress virema [13-15]. Thus, it is important to survey chronic HBV infection among pregnant women and their infants and find other reasons for immunoprophylaxis failure.

There were a total of 4,536 pregnant women aged 16 – 45 years who were enrolled in our study. We showed that the prevalence of HBsAg among pregnant women in Shenyang was 5.49%, which is lower than the overall prevalence of HBsAg among pregnant women in China (8.16%) [16]. The prevalence of HBsAg, anti-HBc and anti-HBs among pregnant women older than 20 years of age was 4.54, 5.69 and 0.61 times that among pregnant women younger than 20 years, respectively. The difference in prevalence of HBsAg, anti-HBc and anti-HBs between pregnant women older than 20 years versus younger than 20 years of age was significant. This could be related to immunoprophylaxis having been universally administered to infants since 1990, which could improve immunity against HBV and decrease HBV infection among their infants.

Among HBsAg positive pregnant women, the majority were HBeAg positive and HBVDNA positive (67.07% and 81.93%, respectively), a minority (14.86%) had a high viral load. Only 12 (4.82%) infants were HBsAg and HBVDNA positive, and their mothers were also HBeAg positive and had HBVDNA>10^7^ IU/ml, which confirmed previous reports. These HBeAg positive and high viral load pregnant women should be offered antiviral treatment during late pregnancy to prevent HBV mother-to-child transmission. There were 2 infants who were both anti-HBs and HBsAg positive. It was reported that the infants were infected with mutant strains which were transmitted from their mothers [17-20]. We should further test sequence analysis to support it and manufacture mutant strain vaccines to prevent HBV mother-to-child transmission.

HBV has been classified into eight genotypes, designated as A-H, based on intergenotypic divergence of at least 8% in the complete nucleotide sequence or more than 4% in the S gene [21-27]. HBV genotypes have distinct geographical distributions and correlate with the severity of liver diseases [28-30]. Are HBV genotypes of great importance in HBV mother-to-child transmission and/or affect the immunization success with HBIG and hepatitis B vaccine? Only genotypes B and C were found among 165 pregnant women. There was a significant difference in genotypes between the pregnant women whose infants were HBV infected and those without HBV infection. Genotype C might be a risk factor for HBV mother to child transmission. It should be recommended that pregnant women who are HBeAg positive and have a high viral load be tested for HBV genotypes.

## Conclusions

There was a significant decline in the HBsAg prevalence among pregnant women and their infants in Shenyang, which might be attributed to universal implementation of immunoprophylaxis since 1990. Genotype C might be a risk factor for HBV mother to child transmission.

## Ethical approval

The Institutional Ethics Committee of Shengjing Hospital of China Medical University approved the study (No.2010PS29K) and the study conformed to the Helsinki declaration of 1996. Informed consent was obtained from all the study subjects prior to the study.

## Abbreviations

(HBV): Hepatitis B virus; (HBsAg): Hepatitis B surface antigen; (HBeAg): Hepatitis B e antigen; (anti-HBs): Hepatitis B surface antibody; (anti-HBc): Hepatitis B core antibody; (HBIG): Hepatitis B immunoglobulin.

## Competing interests

The authors declare that they have no competing interests.

## Authors’ contributions

YD and XGD designed, executed and coordinated the study. YD, QJS and LM contributed in the sample acquirement and laboratory analysis. YD and XGD participated in the drafting of the manuscript and literature search. All authors read and approved the final manuscript.

## References

[B1] Lavanchy D Worldwide epidemiology of HBV infection, disease burden, and vaccine prevention J Clin Virol 2005 34 Suppl 1 S1 S3 16461208 10.1016/s1386-6532(05)00384-7

[B2] Zuckerman JN Zuckerman AJ Current topics in hepatitis B J Infect 2000 41 130 136 11023756 10.1053/jinf.2000.0720

[B3] Michielsen P Van Damme P Viral hepatitis and pregnancy Acta Gastroenterol Belg 1999 62 1 21 29 10333596

[B4] Liang X Bi S Yang W Epidemiological serosurvey of hepatitis B in China–declining HBV prevalence due to hepatitis B vaccination Vaccine 2009 27 47 6550 6557 19729084 10.1016/j.vaccine.2009.08.048

[B5] Cacciola I Cerenzia G Pollicino T Genomic heterogeneity of hepatitis B virus (HBV) and outcome of perinatal HBV infection J Hepatol 2002 36 3 426 432 11867188 10.1016/s0168-8278(01)00295-1

[B6] Papadakis MA Elefsiniotis IS Vlahos G Intrauterine-transplacental transmission of hepatitis B virus (HBV) from hepatitis B e antigen negative (precore mutant, G1896A) chronic HBV infected mothers to their infants, Preliminary results of a prospective study J Clin Virol 2007 38 181 183 17142098 10.1016/j.jcv.2006.10.008

[B7] Zhang SL Yue YF Bai GQ Shi L Jiang H Mechanism of intrauterine infection of hepatitis B virus World J Gastroenterol 2004 10 3 437 438 14760774 10.3748/wjg.v10.i3.437 PMC4724928

[B8] Dong ZQ Zhang XZ Gu XH A randomized controlled trial on interruption of HBV transmission in uterus Chin J Pediatr 2002 40 8 478 480

[B9] Guo Y Liu JQ Meng L Survey of HBsAg-postive pregnant women and their infants regarding measures to prevent maternal-infantile transmission BMC Infect Dis 2010 10 26 20156338 10.1186/1471-2334-10-26 PMC2832640

[B10] Shao ZJ Lei Zhang L Xu JQ Mother-to-infant transmission of hepatitis B virus: a Chinese experience J Med Virol 2011 83 791 795 21360547 10.1002/jmv.22043

[B11] Soderstrom A Norkrans G Lindh M Hepatitis B virus DNA during pregnancy and post partum: aspects on vertical transmission Scand J Infect Dis 2003 35 11–12 814 819 14723355 10.1080/00365540310016547

[B12] Wang Z Zhang J Yang H Quantitative analysis of HBV–DNA level and HBeAg titer in hepatitis B surface antigen positive mothers and their babies: HBeAg passage through the placenta and the rate of decay in babies J Med Virol 2003 71 3 360 366 12966540 10.1002/jmv.10493

[B13] Van Zonneveld M Van Nunen AB Neiesters HG Lamivudine treatment during pregnancy to prevent perinatal transmission of hepatitis B infection J Viral Hepat 2003 10 4 294 297 12823596 10.1046/j.1365-2893.2003.00440.x

[B14] Xu WM Cui YT Wang L Lamivudine treatment in late pregnancy to prevent perinatal transmission of hepatitis B infection: a multicentre, randomized, double-blind, placebo-controlled study J Viral Hepat 2009 16 2 94 103 19175878 10.1111/j.1365-2893.2008.01056.x

[B15] Zhang LJ Wang L Blocking intrauterine infection by telbivudine in pregnant chronic hepatitis B patients Zhonghua Ganzangbing Zazhi 2009 17 8 561 563 19719910

[B16] Centers for Disease Control and Prevention Progress in hepatitis B prevention through universal infant vaccination—China, 1997—2006 MMWR Morb Mortal Wkly Rep 2007 56 441 445 17495790

[B17] Domingo E Quasispecies: concepts and implications for virology Virology 2006 299 17 20

[B18] Domingo E Gomez J Quasispecies and its impact on viral hepatitis Virus Res 2007 127 2 131 150 17349710 10.1016/j.virusres.2007.02.001 PMC7125676

[B19] Gibb R Nimmo GR O’Loughlin P Detectionof HBsAg mutatants in a population with a low prevalence of hepatitis B virus infection J Med Virol 2007 79 4 351 355 17311336 10.1002/jmv.20823

[B20] Gerlich WH Breakthrough of hepatitis B virus escape mutation after vaccination and virus reactivation J Clin Virol 2006 36 Suppl1 S18 S22 16831688 10.1016/s1386-6532(06)80004-1

[B21] Okamoto H Tsuda F Sakugawa H Sastrosoewignjo RI Imai M Miyakawa Y Mayumi M Typing hepatitis B virus by homology in nucleotide sequence: comparison of surface antigen subtypes J Gen Virol 1988 69 2575 2583 3171552 10.1099/0022-1317-69-10-2575

[B22] Norder H Couroucé AM Magnius LO Complete genomes, phylogenetic relatedness, and structural proteins of six strains of the hepatitis B virus, four of which represent two new genotypes Virology 1994 198 489 503 8291231 10.1006/viro.1994.1060

[B23] Kato H Orito E Sugauchi F Ueda R Gish RG Usuda S Miyakawa Y Mizokami M Determination of hepatitis B virus genotype G by polymerase chain reaction with hemi-nested primers J Virol Methods 2001 98 153 159 11576642 10.1016/s0166-0934(01)00374-3

[B24] Arauz-Ruiz P Norder H Robertson BH Magnius LO Genotype H: a new Amerindian genotype of hepatitis B virus revealed in Central America J Gen Virol 2002 83 2059 2073 12124470 10.1099/0022-1317-83-8-2059

[B25] Stuyver L De Gendt S Van Geyt C Zoulim F Fried M Schinazi RF Rossau R A new genotype of hepatitis B virus: complete genome and phylogenetic relatedness J Gen Virol 2000 81 67 74 10640543 10.1099/0022-1317-81-1-67

[B26] Bartholomeusz A Schaefer S Hepatitis B virus genotypes: comparison of genotyping methods Rev Med Virol 2004 14 3 16 14716688 10.1002/rmv.400

[B27] Usuda S Okamoto H Tanaka T Kidd-Ljunggren K Holland PV Miyakawa Y Mayumi M Differentiation of hepatitis B virus genotypes D and E by ELISA using monoclonal antibodies to epitopes on the preS2-region product J Virol Methods 2000 87 81 89 10856755 10.1016/s0166-0934(00)00153-1

[B28] Kao JH Chen PJ Lai MY Chen DS Hepatitis B genotypes correlate with clinical outcomes in patients with chronic hepatitis B Gastroenterology 2000 118 554 559 10702206 10.1016/s0016-5085(00)70261-7

[B29] Kobayashi M Arase Y Ikeda K Tsubota A Suzuki Y Saitoh S Kobayashi M Suzuki F Akuta N Someya T Matsuda M Sato J Kumada H Clinical characteristics of patients infected with hepatitis B virus genotypes A, B, and C J Gastroenterol 2002 37 35 39 11824798 10.1007/s535-002-8130-z

[B30] Ding X Mizokami M Yao G Xu B Orito E Ueda R Nakanishi M Hepatitis B virus genotype distribution among chronic hepatitis B virus carriers in Shanghai, China Intervirology 2001 44 43 47 11223719 10.1159/000050029

